# Understanding the impact of pre-analytic variation in haematological and clinical chemistry analytes on the power of association studies

**DOI:** 10.1093/ije/dyu127

**Published:** 2014-07-31

**Authors:** Amadou Gaye, Tim Peakman, Martin D Tobin, Paul R Burton

**Affiliations:** ^1^Department of Health Sciences, University of Leicester, Leicester, UK and ^2^UK Biobank and University of Manchester, Manchester, UK

**Keywords:** Biobank, Pre-analytical variation, Biosamples, Statistical power

## Abstract

**Background:** Errors, introduced through poor assessment of physical measurement or because of inconsistent or inappropriate standard operating procedures for collecting, processing, storing or analysing haematological and biochemistry analytes, have a negative impact on the power of association studies using the collected data. A dataset from UK Biobank was used to evaluate the impact of pre-analytical variability on the power of association studies.

**Methods:** First, we estimated the proportion of the variance in analyte concentration that may be attributed to delay in processing using variance component analysis. Then, we captured the proportion of heterogeneity between subjects that is due to variability in the rate of degradation of analytes, by fitting a mixed model. Finally, we evaluated the impact of delay in processing on the power of a nested case-control study using a power calculator that we developed and which takes into account uncertainty in outcome and explanatory variables measurements.

**Results:** The results showed that (i) the majority of the analytes investigated in our analysis, were stable over a period of 36 h and (ii) some analytes were unstable and the resulting pre-analytical variation substantially decreased the power of the study, under the settings we investigated.

**Conclusions:** It is important to specify a limited delay in processing for analytes that are very sensitive to delayed assay. If the rate of degradation of an analyte varies between individuals, any delay introduces a bias which increases with increasing delay. If pre-analytical variation occurring due to delays in sample processing is ignored, it affects adversely the power of the studies that use the data.

**Key Messages**

- This work demonstrates that between-subject heterogeneity in the stability of biological analytes can over time lead to substantial degradation
- It also provides a framework for quantifying the power loss due to degradation.

## Introduction

A biobank may be defined as ‘an organized collection of human biological material (e.g. blood, urine or extracted DNA) and associated information stored for one or more research purposes’.^1^^,^^2^ Most contemporary biobanks are large by design because the aetiological determinants(genes, environment and interactions) of complex diseases are typically weak (e.g. relative risks between 1.1 and 1.3) and their resolution therefore demands many subjects^3^ with data that are both accurate and precise. Crucially, errors introduced through poor assessment of physical measurement or because of inconsistent or inappropriate standard operating procedures (SOPs) for collecting, processing, storing or analysing biosamples can seriously impair data quality. This can dramatically reduce the statistical power of a study, particularly if one is studying gene-environment interactions.^3^^,^^4^ Given the vast cost and effort that are needed to establish and maintain a contemporary biobank, even a small loss of power can impact substantially on the balance of costs and benefits of developing adequately powered study resources. The quality and future utility of biological samples can be affected by factors arising during the collection, transport, processing and storage of biosamples.^5^ It is therefore crucial to use carefully selected and validated protocols that minimize any changes in the quantity or nature of the constituents (biological analytes) of each biosample and allow for the further re-use of the samples.^6–9^ It is for this reason that certain SOPs^9^^,^^10^ that ensure minimal pre-analytical variability between samples are published—as best practice guidelines for biological resource centres—by organizations involved in the conceptualization, design and conduct of sample collection, processing and analysis. This includes guidelines from the National Cancer Institute (NCI),^11^ Organization for Economic Cooperation and Development (OECD)^12^ and International Society for B, Environmental R (ISBER),^13^ as catalogued in the website of the Public Population Project in Genomics (P^3^G).^14^^,^^15^

A critical issue is the impact of any delay between sample collection and the processing step that definitively stabilizes that sample (the ‘needle-to-freezer’ time). This is because some biological analytes are not stable before permanent storage and their concentration changes over time. For example, the concentration of aspartame transaminase (AST), a biochemical analyte present in red blood cells, increases by 15.2% at 21°C and by 1.5% at 4°C, over 24 h.^16^ If there is any tendency for an analyte to degrade, or accumulate, over time ahead of stabilization, any delay in definitive processing will introduce measurement error.

If the rate of degradation is very similar in all samples, then an SOP requiring a fixed (though non-zero) delay till processing (e.g*.* 24 h) will ensure that all samples to be analysed will be similarly affected and biostatistical and/or epidemiological analyses may be unbiased. But if case and control samples are processed under different protocols—e.g. with a different needle-to-freezer time—serious systematic bias may arise. Additional problems will arise if the rate of degradation varies markedly from subject to subject. Then any delay in processing will introduce random error that will reduce statistical power, even if every sample is subject to the same delay. Furthermore, the magnitude of the consequent bias will become steadily more serious as the duration increases.

There are three possible solutions to pre-analytical variability in this setting: (i) use a common standard operating procedure (SOP) involving local processing at all collection sites; (ii) set up a large study with rapid sample transportation and central processing such that any delays are minimal; or (iii) carefully assess the impact of biosample deterioration so that an evidence-based decision can be made regarding the maximum delay that may be reasonable and that, where possible, the delay time can be taken into proper account in designing or analysing studies to be based on the stored biosamples.

All else being equal, an SOP involving a short needle-to-freezer time in all participants is undoubtedly to be preferred. But the first two solutions are both expensive. For example, the first solution requires every collection site to have a local capacity for state-of-the-art processing and storage, rather than restricting such facilities to a single central facility. In a nationwide study this may well be unaffordable. Given that any decisions about the optimum protocol will therefore have major scientific and financial implications, it is clear that a sound quantitative understanding is required of the manner in which analytes degrade or accumulate in unprocessed samples. But, because the degradation profile is likely to vary from analyte to analyte, it is necessary to investigate a wide range of different biomarkers.

The analyses described in this article fulfil these aims. They form part of the international biobank harmonization programmes of P^3^G, Maelstrom^17^ and BioSHaRE-EU.^18^ The work is based on a set of pre-pilot blood samples that were originally collected by UK Biobank with the express purpose of exploring the stability of analytes in the period prior to definitive storage. Although these data have previously been analysed^19^ with a closely related intention in mind, the conclusions of those earlier analyses focused primarily on the rate of degradation/accumulation per se, rather than on the potential impact of heterogeneity in rate among samples from different participants. As shall be demonstrated, a comprehensive exploration of the latter raises important additional considerations that ought to be taken into proper account in developing the SOPs for sample collection in a major biobank and/or in designing or analysing the individual studies to be based upon a biobank.

## Methods

Three complementary sets of analyses were undertaken, exploring changes in the concentration of 47 analytes over time in the period before definitive long-term storage. The first set explored the extent to which any variation in the measured concentration of each analyte might reasonably be attributed to delays in processing. These replicated the equivalent analyses reported in the earlier paper^19^ by Jackson *et al.* and demonstrated that our approaches were fundamentally equivalent. The second set focused specifically on heterogeneity in the rate of change of concentration of each analyte, between samples from different subjects. The third set explored the impact of that heterogeneity on the power of a typical association study. It is in the interpretation of the second and third sets of analyses that our investigation moves beyond that reported by Jackson *et al.*^19^ and leads to important additional conclusions that should necessarily be taken into account in considering the power of association analyses to be based on a biobank.

### Description of the data

UK Biobank (UKBB) is a large biobank of 500 000 participants, aimed at investigating the role of genetic factors, environmental exposures and lifestyle in the causes of major diseases of late and middle age^20^. The data used in our analysis are from a pre-pilot study set up during the design phase of UKBB. They consist of the measured values of 47 blood and urine analytes from 40 subjects, that were put into definitive long-term frozen storage between 0 and 36 h after initial blood collection. All samples were kept at 4°C until they were frozen. The 40 samples are from healthy unrelated volunteers that were not among the 500 000 participants ultimately recruited into UK Biobank. The 40 volunteers consisted of 20 males with an average age of 56 years and a median of 56 years, and 20 females with an average age of 51 years and a median of 49 years. In comparison, the UK biobank participants were from males and females aged 40–69 years;^21^ the proportions of males and females among the 500 000 UK Biobank participants are, respectively, 45 and 55%.^22^ We do not have information about the socio-economic statuses of the 40 subjects who provided the samples analysed in this work.

**Figure 1.**
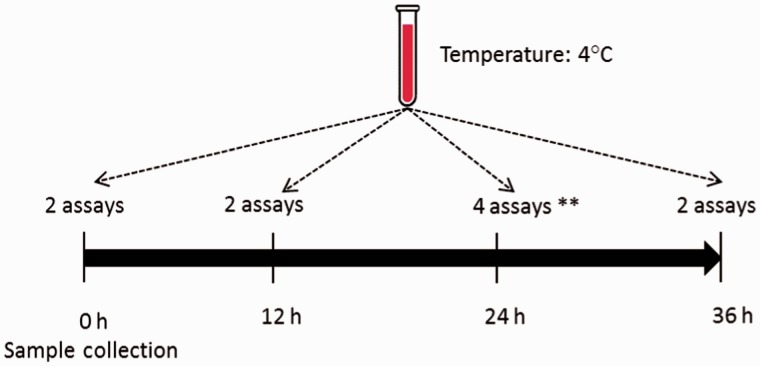
Repeated measurements are taken over 24 or 36 h for each analyte and each subject. **Two assays only used in the present analysis.

The data structure is hierarchical, with three levels: subject; time point; and replicate measures. Analyte concentrations were measured at four time points (0, 12, 24 and 36 h) for 19 of the 47 analytes and at two time points (0 and 24 h) for the other 28. The designated times represent time since sample collection; 0 h implies the assay was carried out immediately after sample collection (Figure 1). Two replicate measurements were taken at each nominal time point except at 24 h when four replicate measurements were taken: two of those four were used to study the effect of freeze/thaw.^19^ These two latter samples were not true replicates and were excluded from our analysis. A total of 320 measurement values were therefore analysed across the 40 participants for each of the 19 analytes with measurement at four distinct time points and 160 values in the other 28 analytes. The analyte C Reactive Protein was excluded from the analysis because its data were censored; all measurement values <0.2 were reported as 0.2, and this distorted the correlation structure both within and between subjects. The remaining 46 analytes were analysed one at a time.

### First set of analyses: estimating the proportion of the variance in analyte concentration that may reasonably be attributed to delay in processing

**Figure 2.**
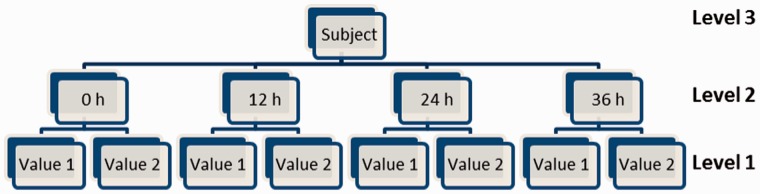
Graphical depiction of a three-level model fitted in MLwiN. 0 h and 24 h components only for analytes measured twice (i.e. two replicates).

The observed variability between different samples from different participants reflects a combination of: (i) real biological heterogeneity between subjects; (ii) random measurement error; and (iii) pre-analytic variability caused by processing delays. It is the first of these that carries the information driving useful scientific investigation. Although the variability due to delay in processing is in fact a ‘real’ biological effecti.e. the change in concentration (usually degradation) of a biological analyte over time isa real phenomenon, its effect is to produce a loss of information from the sample and, in that sense, is analogous to random measurement error. To estimate the proportion of the observed variability between subjects that can reasonably be attributed to delay in processing, a three-level variance component model (Figure 2) was fitted in MLwiN 2.1.^23^

This is a simple ‘random intercept’ model:
Model 1
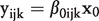

Where i, j and k, respectively, index observations at the replicate measurement, processing time and subject levels of the data hierarchy, 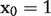 and 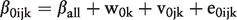. The terms 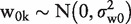, 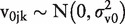 and 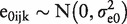 are random ‘errors’acting at different levels of the hierarchy. Variation at level 1 (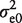) reflects random measurement error. Variance at level 2 (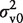) incorporates variability arising from delay in processing. Level 3 is the subject level and 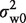 therefore captures the real biological difference between subjects that provides the basis of useful scientific enquiry. The distributions of the residuals at levels 1, 2 and 3—e_0ijk_, v_0jk_ and w_0k_—were explored and verified as Gaussian using normal probability plots. The model was fitted without explanatory variables; the ‘intercept’ covariate, x_0_, took the constant value 1. The response of each observational unit (each sample in every subject) was therefore modelled as having a common underlying expectation, β_all_.

Under the stated parameterisation, time was effectively modelled as a non-ordered categorical variable. An ordinal parameterization could instead have been used. This would have been more powerful for detecting a weak but consistent decline (or increase) in concentration over time. But, for this part of the analysis, it was considered preferable to minimize any assumptions and to treat each time point as an independent entity rather than assuming a natural order. Given that consistent changes over time were easily detected for many of the analytes with four time points anyway, it would appear that this decision was reasonable. For analytes measured at only two time points, the non-ordered and ordinal models are equivalent.

### Second set of analyses: investigating between-subject heterogeneity in the rate of decline(or accumulation) of analytes

**Figure 3.**
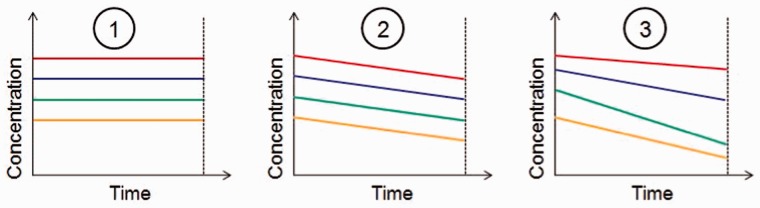
Variation in biological analyte concentration over time; changes in concentration of one analyte in four individuals are compared in three different scenarios.

**Figure 4.**
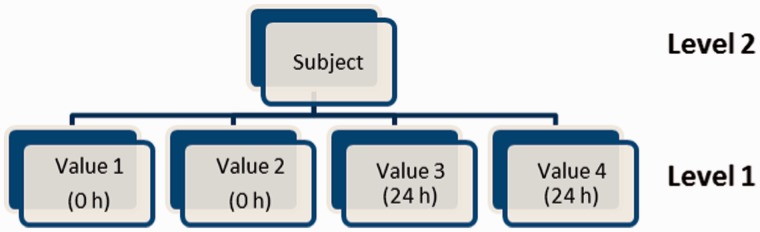
Graphical depiction of a two-level multilevel model for an analyte measured at two time points (0 and 24 h) (model incorporates a time covariate).

In considering the stability of a biological analyte over time, it is helpful to recognize three fundamental possibilities (Figure 3): (i) samples are stable and there is no change in the concentration of the analyte over time; (ii) samples are unstable, but the rate of change in concentration is the same in all samples from all individuals; and (iii) samples are unstable and the rate of change in concentration varies from individual to individual. These three scenarios may be modelled using a series of nested two-level multilevel models. Figure 4 depicts the model for analytes with two measurements at 0 h and 24 h. For analytes with measurements at 0 h, 12 h, 24 h and 36 h, there would be four additional boxes at level 1 (two replicate measures at each of 12 h and 36 h) and the dummy time covariate is parameterized as indicated below.

Scenario 3 is the most general case. It is encapsulated in the model 2 below:
Model 2
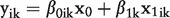

where i and k index individual measurements and different subjects, respectively:

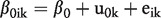


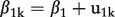


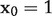


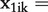
a dummy time covariate denoting the timing of the i^th^ measurement in the k^th^ subject. This covariate is coded as 0 and 1 respectively for the time points 0 h and 24 h (for analytes with two measurements); and as 0, 0.5, 1 and 1.5 respectively for the time points 0 h, 12 h, 24 h and 36 h (for analytes with four measurements).

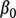
= expected concentration of analyte at time point 0 h;
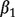
= expected change in concentration of analyte between time points 0 h and 24 h;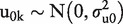 subject level random effect reflecting the variance between subjects in the expected concentration of the analyte at time point 0 h;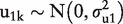 Ssubject level random effect reflecting the variance between subjects in the expected rate of change in concentration between time points 0 h and 24 h;
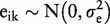
 independent residual error terms at level 1.

Under the most general model (scenario 3), the expected change in concentration between time points 0 h and 24 h may be non-zero (β_1_ ≠ 0) and the rate of change may vary from subject to subject (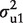 > 0). Scenario 2 is a special case of scenario 3, in which the slope may be non-zero (β_1_ ≠ 0) but the rate of change is the same in samples from all subjects 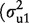 = 0). Scenario 1 is a special case of scenario 2, in which the slope is identically zero in all subjects (β_1_ = 0 and 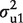 = 0). Statistical inferences may be based on a comparison of the likelihood of the nested models. For example, a comparison of the likelihood between scenarios 3 and 2 enables inference on the question: ‘Is there significant evidence of between-subject heterogeneity in slope?’

The generality of model 2 may now be usefully extended to allow a correlation between 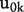 and 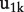, reflected in an additional term (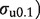 denoting the covariance between the two sets of random effects. These estimated variance and covariance terms might then be used to estimate the overall variance at 0 h and the overall variance at 24 h:
Equation 1
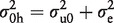

Equation 2
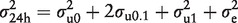

All models used in undertaking the second set of analyses were fitted using MLwiN^23^.

### Third set of analyses: estimating the impact of delays in sample processing on the power of case-control studies

The second set of analyses (above) would appear to indicate a straightforward way to address the scientific aims of the third set of analyses. Our results for the second set of analyses are similar to those of Jackson *et al.* who concluded that the contribution of heterogeneity in slope was negligible, and this supported their general conclusion that ‘any instability in assay results up to 36 h is likely to be small in comparison with between-individual differences and assay error’.

**Figure 5.**
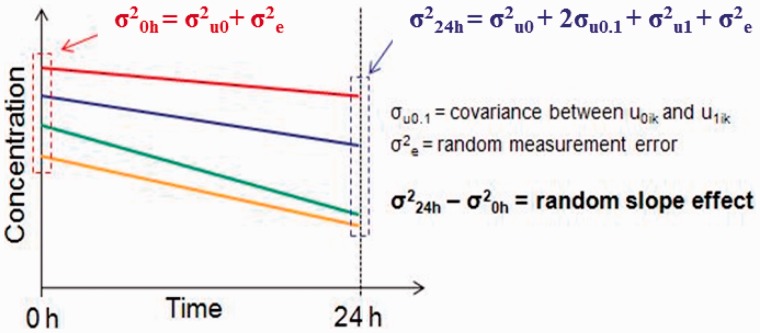
Using the variance components to determine total variability at 0 and 24 h.

However, Jackson *et al*.’s ‘percentage of total variation in assay measurements explained by between-individual differences’ is a blunt tool. The between-individual variance at baseline (0 h) reflects the true biological signal of interest. Any additional variance arising, after 0 h, from between-subject differences in slope, reflects a distortion of that true signal. Furthermore, after baseline, the covariance between the baseline biological signal and the subsequent slope complicates the interpretation of the combined ‘between-individual variance’. This is because two additional terms contribute to between-individual variance after time 0: 
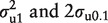
 (Equations 1 and 2). If the covariance term 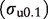 is negative, a higher analyte concentration at baseline is, on average, associated with a steeper subsequent decline. Lines depicting concentration (e.g. Figure 5) may therefore converge from left to right but, despite the resultant fall in total variability, the true biological signal reflected in the magnitude of 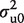 nevertheless degrades as time progresses. Arguing quantitatively, if 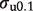 is negative and greater in absolute magnitude than 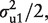 the total variance will decline, but the biological signal will nevertheless degrade and statistical power will fall.

One situation is particularly illuminating: if 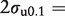 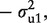 the total between-individual variance at time 0 and time 1 (24 h) will be equivalent: 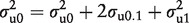, as will the total variance 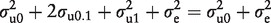. The proportion of total variability that is explained by variability between individuals—the parameter that Jackson *et al.* compare over time—therefore remains constant, but the original biological signal has degraded and is partially balanced by variance arising from between-subject heterogeneity of slope. This latter may well be a real biological difference between individuals, but it will not in general be the same biological difference that accounts for between-subject heterogeneity at baseline. Thus, although Figure 2 in Jackson *et al.*^19^ does indeed demonstrate that the proportion of total variance that can be explained by variation between individuals is relatively constant over time for most analytes, slope heterogeneity between individuals may nevertheless be causing a substantial degradation of the baseline biological signal and a loss of statistical power. An alternative approach is therefore needed to explore the degradation more rigorously: a direct comparison of statistical power with and without slope heterogeneity between individuals.

To illustrate, let us consider perhaps the simplest of scenarios: a nested case-control study is to focus on the question: ‘Is the concentration of analyte A associated with disease D?’ To explore statistical power, we might use the ESPRESSO simulation-based power calculator (Estimating Sample-size and Power in R by Exploring Simulated Study Outcomes), that was originally developed to estimate the power of nested case-control analyses based on UK Biobank^24^ and has since been extended to model quantitative exposures.^25^^,^^26^ The use of ESPRESSO to estimate the power of case-control studies using unconditional logistic regression has been described in detail elsewhere.^3^

Briefly, a simulation-based power calculation may be undertaken involving a logistic regression model taking case-control status (presence or absence of disease D) as its binary outcome, one quantitative covariate reflecting the analyte of interest A, and which has been ‘spiked’ by incorporating realistic levels of measurement error, and other factors that are likely to influence statistical power (see Appendix A, available as Supplementary data at *IJE* online). The required sample size for a case-control analysis to have 80% power to detect the true association between D and A (across a range of true effect sizes) can then be compared between two different settings. All simulation parameters pertaining to the generation of A_O_, the observed analyte concentration, in both settings for each analyte are derived directly from the multilevel models fitted earlier in the analysis.

#### Setting 1—no heterogeneity in degradation slope

All measurements are assumed to have been taken at 0 h and slope heterogeneity has no impact. A vector of ‘true’ simulated analyte concentrations A_T_ (error-free data) is generated using a two-step procedure that separately generates the fixed expected values (A_F_) and random terms reflecting biological heterogeneity (A_R_). A_T_ is generated with a mean β_0_ (expected intercept at time 0 h) and a between-subject variance of 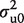 (both estimated in the MLwiN analysis for the chosen analyte—Table 2). The ‘observed’ analyte concentration A_O_ is then obtained by adding a random measurement error term (E_R_) to A_T_:

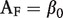


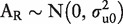


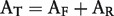


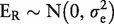


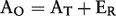

In this particular setting, the true biological signal associated with the analyte is captured by variation in A_T_ and the only error (measurement error)is reflected in 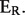 As in any ESPRESSO analysis,^3^ 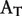 is used to stochastically generate a vector of simulated ‘true’ case-control statuses 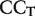, which are then subject to appropriate misclassification error to generate an ‘observed’ case-control status vector 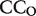. A logistic regression of 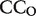 on 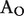 now allows an estimation—based on this single simulation—of the odds ratio (and standard error) relating 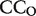 to a one unit change in 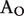, and this in turn allows an empirical estimate of the sample size that would have been required to detect this effect with 80% power.^3^ This simulation process is then repeated 500 times, and the estimated sample sizes averaged to generate a consistent estimate of the required sample size.^3^

#### Setting 2—slope heterogeneity present

**A**ll measurements are taken at 24 h. Precisely the same procedure is followed as in setting 1, except: (i) the fixed effects include 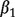, the overall slope, which is a fixed value in the simulation and has no impact on power; (ii) the random effects include S_R_ which reflect slope heterogeneity; (iii) the A_R_ and S_R_ random effects are generated so as to have the correct covariance (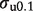) (Equation 2) using an approach we originally developed for simulating—potentially negatively correlated—family data.^27^^,^^28^ Appendix B provides R code for generating the required covariance (available as Supplementary data at *IJE* online).

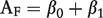


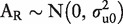


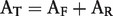


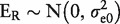


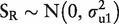



      Crucially: Cov(A_R_, S_R_) = 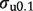


      And finally: 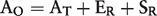


It should be noted that S*_R_*, the random effects reflecting slope heterogeneity, are actually multiplied by ‘time’ (see model 2), but the parameterization is such that at 24 h time = 1, so it is excluded from the presentation for simplicity.

Having estimated the sample sizes required in both settings, their ratio (setting 2/setting 1) indicates the multiplicative increase in sample size that is required to precisely compensate for slope heterogeneity if sample processing is delayed to 24 h.

In order to render the power calculations for each analyte directly comparable, all distributional parameters—as estimated in MLwiN—were first standardized by dividing all the variance components for a given analyte by its estimated 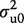, subtracting the mean (β_0_), and dividing β_1_ by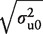. This meant that the estimated sample size provides 80% power to detect the true impact (as simulated) of one standard deviation change on the odds of the particular disease being studied.

As usual,^3^ the ESPRESSO analyses incorporated a full and realistic range of power-determining factors (Appendix A, available as Supplementary data at *IJE* online; see online material^25^^,^^26^ for a full list of parameter values that were actually specified).

## Results

### First set of analyses

Using the three-level model with no heterogeneity in slope between subjects (Model 1), the proportion of the observed variability that can reasonably be attributed to delay in processing time (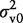) is ≥10% for 16 out of 46 analytes (Table 1). Eight analytes have 5–10% of the observed variability attributable to delay in processing. For the remaining 22 analytes, the contribution of the variance resulting from delay in processing represents less than 5% of the observed variance. Bicarbonate seems particularly sensitive to delayed processing, with 61% of the observed variability coming from 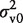.

**Table 1.** Analytes ranked by decreasing contribution of delay in processing to observed variability between subjects. Two replicate measurements where available for the analytes highlighted in light and four for the analyte highlighted in dark

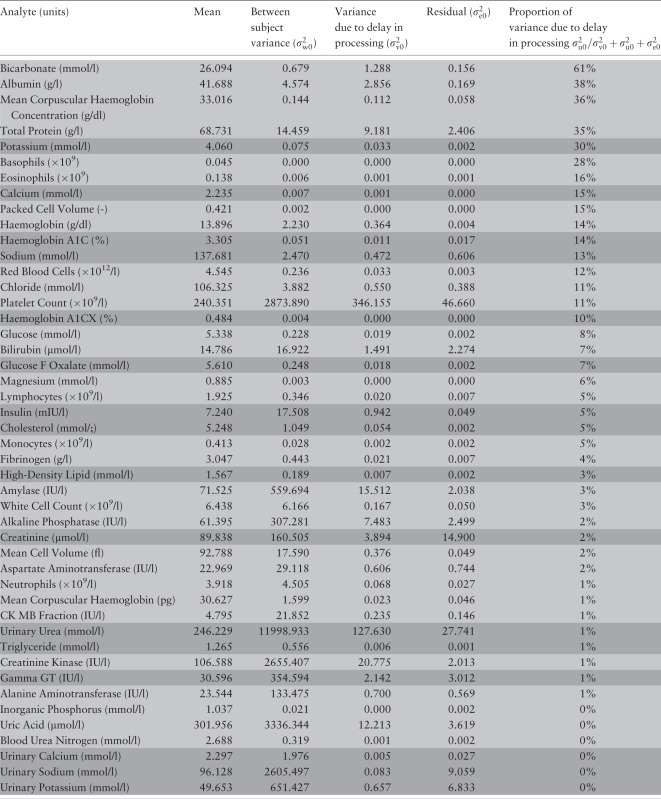

### Second set of analyses

Results are summarized in Table 2. Crucially by applying the likelihood ratio test to Model 2 (Figure 2), a comparison under scenarios 2 (constant slope across individuals) and 3 (heterogeneity of slope across individuals) indicates that almost all analytes exhibited significant evidence of between-person heterogeneity of slope. This finding is entirely consistent with the equivalent finding reported by Jackson *et al.*^19^ However, they concluded that despite the statistical significance of this slope heterogeneity, it would in practice have little impact on statistical power.

**Table 2.** Heterogeneity in rate of change of analyte concentration (slope variance) and results of the comparison between the models for scenario 3 and scenario 2 (i.e. 2-level model with and without a random slope). The analytes for which the models were fitted with two time points are highlighted in light whereas those for which the models were fitted with four time points are highlighted in dark

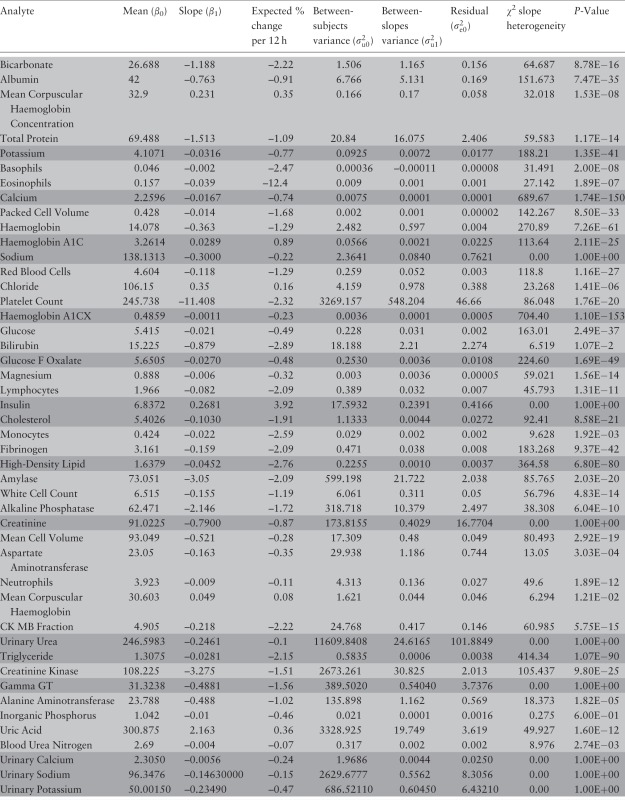

### Third set of analyses

The quantitative impact of slope heterogeneity on estimated sample size requirement is detailed in Table 3. Five analytes demand the sample size to be more than doubled: bicarbonate (2.32), albumin (2.27), total protein (2.21), basophils (2.19) and magnesium (2.15). Three further analytes required sample size inflation of at least 25% and, in total, 29 analytes demand an increase of at least 10%. As would be anticipated, the loss of power, and hence the required increase in sample size, is largest when 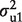 (the slope heterogeneity) is large relative to the biological signal (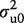).

**Table 3.** Sample size increase required to compensate for power loss caused by bias arising from slope heterogeneity. The analytes for which the models were fitted with two time points are highlighted in light whereas those for which the models were fitted with four time points are highlighted in dark

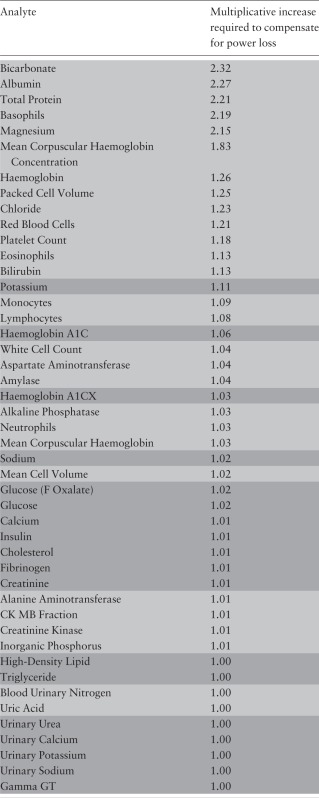

## Discussion

Because of the complexity of the aetiological architecture of complex diseases, and because individual causal effects are often weak, statistical power is at a premium. This implies a need for large sample sizes and measurements that are both accurate and precise.^3^^,^^29^ Standard operating procedures (SOPs) for biosample collection, processing, storage and analysis must be explored in detail and chosen with care. Furthermore, key decisions about SOPs that are taken when a biobank or major epidemiological study is first set up have an irrevocable impact on the future utility of data and samples. They not only impact on the future science to be based on a large-scale biobank, but also on utility of the huge investment that societies and science policy makers are currently willing to make in creating and managing biobanks. The particular set of SOPs that is adopted by a biobank really matters.

The work reported in this paper builds directly on an earlier paper by Jackson *et al*.^19^ Both papers use empirical pre-pilot data collected by UK Biobank to explore one key element of the SOP for biosample handling—the impact of extending the needle-to-freezer time, i.e. the time delay between collecting a biosample (blood or urine) and putting aliquots of that sample into definitive storage. Our paper significantly extends the interpretation of one critical parameter in the model: the impact of between-subject heterogeneity in the rate of degradation (decline or accumulation) of each analyte studied. This extended interpretation substantively changes the conclusions of the overall analysis, and must therefore be taken into account when designing or using a biobank.

Our analyses and those undertaken in the earlier paper^19^ can be directly compared; the mathematical models are equivalent and quantitative conclusions are effectively the same. Thus, the estimated average percentage change for each analyte over 12 h, the estimated probability that a trend over time is truly negative and the likelihood ratio test for heterogeneity are all very similar. For example, Jackson *et al.*^19^ reported that the percentage change in analyte concentration over 12 h of delay was <3% in absolute magnitude for all but two analytes: insulin (+3.9%) and eosinophil count (−12%). We entirely concur, with corresponding estimates of +3.92% and −12.4% for insulin and eosinophil count, respectively.

Where our interpretation departs from the Jackson *et al*. paper,^19^ and our analyses and findings provide an important complement to theirs, is in regard to the impact of heterogeneity between individuals in the rate of degradation of a given analyte. On the basis of their Figure 2 (‘Percentage of total variation in assay measurements explained by between-individual differences, plotted against time of measurement’), Jackson *et al.*^19^ concluded that any such heterogeneity would have only a limited impact. This contributed to a globally reassuring conclusion that ‘any instability in assay results up to 36 h is likely to be small in comparison with between-individual differences and assay error’, and that ‘a single assay measurement at any time between 0 and 36 h should give a representative value of the analyte concentration at time zero for that individual’.

In contrast, our extended analysis suggests that the situation is more complex. This is of direct relevance both to selecting SOPs for biosample management in designing and constructing a biobank, and to designing and choosing the sample size of future studies that may use that biobank as a platform for investigating causal pathways involving biomarkers and complex diseases. Thus, our analyses suggest that for five of the biomarkers we studied (more than 1 in 10), the loss of biological signal consequent upon delayed processing for even 24 h would require at least a doubling of the sample size of a case-control study to investigate the impact of that analyte on disease risk. More broadly, our results suggest that between-subject heterogeneity in the stability of a significant number of key analytes can, over 24 h, lead to substantive degradation in the biological information in biosamples and a requirement to increase power by at least 25% to compensate (Table 3). This interpretation implies that—if such analytes are to be explored in a study based on a biobank—there is a need to take proper account of the processing delay in interpreting the measured concentrations of those analytes, and to appropriately increase the sample size of the study to counter this loss of information.

An important general message is that the management rigor, including forward planning and comprehensive documentation, that is fundamental to setting up and exploiting a large scale biobank,^15^ is desirable in its own right. Well-thought-through and well-documented SOPs, appropriate ancillary data (e.g*.* recording just how long an individual biosample was delayed before entering definitive frozen storage) and formal tests of quality assurance are all critical if we are to know where problems may arise and can respond appropriately to them. Furthermore, given that all good biobanks do implement a rigorous regime for data and sample management, the larger the size of any given biobank the better, provided this does not impinge on biosample quality. It is often impossible to avoid combining scarce data from several studies together in order to answer an important scientific question,^3^ but there is an undoubted benefit in minimizing the number of different studies that must be combined—because this limits the potential heterogeneity. Ultimately, one can do no more than aim to work where possible with larger, well designed biobanks/studies, and to use whatever ancillary information may be available from each to try to control for whatever heterogeneity may be unavoidable.

Large-scale biobanks—particularly those with a national or even supranational design —must carefully think through their SOPs for obtaining, transporting and processing biosamples. Given that some analytes do appear to be particularly sensitive to a prolonged needle-freezer time, and given that in some cases this is due to heterogeneity in degradation slope, this delay should wherever possible be minimized. However, immediate biosample processing at point of collection can be very expensive; some delay is therefore inevitable and an undesirably long delay may sometimes be unavoidable. It is therefore universally desirable to record that delay, to provide those ancillary data with the relevant biosamples and/or analyte concentration estimates when they are released to researchers and to ensure that the time course of pre-processing-related degradation of commonly studied analytes is properly understood.

Our study complements the findings of Jackson *et al.* in identifying a number of analytes where the rate of degradation is unusually high. Particular care should be taken in analysing data pertaining to such analytes if cases and controls have been sourced from different studies with different SOPs or if the delay times vary widely within an individual study. In some circumstances, where one of these analytes happens to be the primary focus of scientific attention, and there is a desire to avoid having to adjust for differing delay times, consideration maybe ought to be given to delaying processing of all samples to a common, achievable, goal. However, our analysis suggests that such a plan may be counterproductive if the analyte happens to be one of those where the heterogeneity of degradation varies so much between samples that any delay can lead to loss of power.

Finally, if an analyte of interest is known to be particularly sensitive to processing delay—either because of rapid degradation or because of marked heterogeneity of degradation—full account must be taken in designing sub-studies. Rather than trying to produce exhaustive lists of power adjustments for different delay times for a wide range of analytes, we are currently extending the ESPRESSO power calculator to take data from studies such as the biobank pre-pilot study analysed in this paper, and to convert these data into sample size inflation figures for different settings and analytes. Given that there are now many more analytes that can reasonably be analysed in a high throughput manner than there were when the UK Biobank data that we analysed were collected, there is a clear need to undertake equivalent studies for other analytes and in other settings. For robustness, these studies should be based on measurements at four time points for all analytes rather than just two for some.

Although the data used in this project were generated in a pre-pilot phase of UK Biobank, the nature of the analysis is such that its conclusions should be generalizable to other biobanks and large-scale biosample collections. Furthermore, although UK Biobank has now completed its primary sample collection and its standard operating protocols for that collection are therefore immutable, the analysis we describe will be of value to UK Biobank in future sample collection sweeps, and to potential users designing sub-studies to explore the effect of biomarkers.

## Supplementary Data

Supplementary data are available at *IJE* online

## Funding

The initial development of the ESPRESSO tool used in this work was supported by the Canadian Institutes of Health Research through the Public Population Project in Genomics P^3^G [grant number 64070822]. The current development of ESPRESSO is funded by the European Union through the Biobank Standardisation and Harmonisation for Research Excellence in the European Union (BioSHaRE-EU) project [FP7-HEALTH-F4-201433]. Martin D Tobin holds a Medical Research Council Senior Clinical Fellowship (G0902313). This report presents independent research funded partially by the National Institute for Health Research (NIHR). The views expressed are those of the author(s) and not necessarily those of the NHS, the NIHR or the Department of Health.

## Supplementary Material

Supplementary Data
