# Supplementary material for: Understanding the impact of pre-analytic variation in haematological and clinical chemistry analytes on the power of association studies
Source: Int J Epidemiol. 2014 Jul 31;43(5):1633–44. doi: 10.1093/ije/dyu127 (PMC4190517; doi:10.1093/ije/dyu127)
Supplement: Supplementary Data [file supp_43_5_1633__index.html]

Understanding the impact of pre-analytic variation in haematological and clinical chemistry analytes on the power of association studies — Understanding the impact of pre-analytic variation in haematological and clinical chemistry analytes on the power of association studies — Supplementary Data 

# Understanding the impact of pre-analytic variation in haematological and clinical chemistry analytes on the power of association studies

## Supplementary Data

files

**Files in this Data Supplement:**

- Supplementary Data - docx file
