# Supplementary material for: Understanding the impact of pre-analytic variation in haematological and clinical chemistry analytes on the power of association studies
Source: Int J Epidemiol. 2014 Jul 31;43(5):1633–44. doi: 10.1093/ije/dyu127 (PMC4190517; doi:10.1093/ije/dyu127)
Supplement: Supplementary Data [file supp_dyu127_ije-2013-08-0779-File002.docx]

# APPENDIX

## Input parameters for the sample size calculations in the third set of analyses

The power calculations were carried out using the below parameters for the five different odds ratios (OR of the environmental determinant) reported in the table. The sample size multiplicative increase values reported in Table 3 are the average multiplicative increase calculated across the five ORs.

| **PARAMETER** | **DESCRIPTION** | **VALUE** |
| --- | --- | --- |
| **General and simulation parameters** |  |  |
| *number of runs* | an integer that indicates the number of runs | 500 |
| *number of cases* | an integer indicating the number of cases for a binary outcome model | 1000 |
| *number of controls* | an integer indicating the number of controls for a binary outcome model | 4000 |
| *p.value* | a value defining statistical significance | 1.00E-04 |
| *power required* | a value between 0.1 and 1defining the desired level of statistical power | 0.8 |
| *baseline OR* | the effect associated with the heterogeneity in baseline disease risk for a binary outcome | 12.36 |
| **Parameters for the outcome variable** |  |  |
| *outcome model* | a binary indicator variable set to 0 if the outcome is to be modelled as binary and 1 if it is to be modelled as normally distributed | 0 |
| *disease prevalence* | the frequency of the binary outcome in the general population | 0.1 |
| *sensitivity* | the sensitivity of the assessment of the outcome | 1 |
| *specificity* | the specificity of the assessment of the outcome | 1 |
| **Parameters for the environmental determinant** |  |  |
| *exposure model* | a discrete indicator variable set to 0 if the exposure is binary, 1 if the exposure follows the normal distribution and 2 if it follows the uniform distribution. | 1 |
| *OR* | the frequency of the ‘at risk’ environmental exposure in the study population | 1.1, 1.3, 1.5, 2.0, 2.5 |
| *reliability* | reliability of the measurement of the quantitative ‘at risk’ environmental exposure | 1 |

## The R code for generating the required covariance in section 3.3

#The purpose of this code is to demonstrate that the way we are simulating #the elements required to produce the sample size calculations results #reported in section 3.3 does generate the right covariance structure. The #difference in sample size requirement between the two settings (samples #processed right after collection or with some delay) explained in section

#2.3 of the methods, is solely due to slope heterogeneity, because it is #only the slope variance and the covariance between intercept and slope #that are different between the two settings. This code shows that the #covariance we obtained empirically in our simulations is the same as the #covariance obtained theoretically, hence confirming that the data we #simulated have the correct characteristics.

########################################################

# STEP 1 #

########################################################

# SET SEED AND NUMBER OF OBSERVATIONS

set.seed(149793)

sampsize.espresso <- 1000000

# MLwiN DERIVED PARAMETERS TO SIMULATE (HCO3 example)

beta0 <- 26.688

beta1 <- (-1.187)

s2U0 <- 1.50550

s2U1 <- 1.16480

covU01 <- (-0.47420)

s2E <- 0.15630

abscov <- abs(covU01)

########################################################

# STEP 2 #

########################################################

# ALLOW FOR COV > abs(s2U0) or abs(s2U1)

# START BY IDENTIFYING LARGEST RATIO

rat0 <- 1

if(abscov > s2U0) {rat0 <- sqrt(s2U1/abscov)}

# USE THE HEAD SPACE PROVIDED BY s2U1 BEING GREATER THAN abscov

rat1 <- 1

if(abscov > s2U1) {rat1 <- sqrt(s2U0/abscov)}

# USE THE HEAD SPACE PROVIDED BY s2U0 BEING GREATER THAN abscov

# maxrat IS LARGER OF THE TWO RATIOS

# SHRINK FACTOR ENSURES THAT BOTH VARIANCES ARE VERY SLIGHTLY

# LARGER THAN VARIANCE CORRECTION TO GENERATE COVARIANCE

shrink <- 0.995

maxrat <- (max(c(rat0,rat1)))^shrink

# CALCULATE KEY VARIANCES TO MODEL

# VARIANCE AT T0 WITH MEASUREMENT ERROR

varT0 <- s2U0 + s2E

# VARIANCE AT T1 WITH ALL ERRORS

varT1 <- s2U0 + s2U1 + 2*covU01 + s2E

# VARIANCE AT T0 WITHOUT MEASUREMENT ERROR

varT0.noE <- s2U0

# VARIANCE AT T1 WITH SLOPE HETEROGENEITY BUT NO MEASUREMENT ERROR

varT1.noE <- s2U0 + s2U1 + 2*covU01

# COVARIANCE BETWEEN ANALYTE LEVELS AT T0 AND T1

covT0T1 <- s2U0 + covU01

# GENERATE RANDOM EFFECTS (REs) TO BE SHARED TO GENERATE CORRECT COVARIANCE STRUCTURE

# START BY USING ABSOLUTE VALUE OF ORIGINAL COVARIANCE

cov.element <- abscov

# GENERATE BASELINE REs USING THIS AS THE VARIANCE OF GENERATION

element <- rnorm(sampsize.espresso,0,sqrt(cov.element))

# DEAL WITH NEGATIVE vs POSITIVE COVARIANCES FOR INTERCEPT:SLOPE

mult.sign <- (1 - 2*(covU01 <0 ))

if(s2U0 >= s2U1)

{

element.00 <- element*maxrat

element.11 <- (element/maxrat)*mult.sign

cov.element.00 <- cov.element*(maxrat^2)

cov.element.11 <- cov.element/(maxrat^2)

}

if(s2U0<s2U1)

{

element.00 <- element/maxrat

element.11 <- (element*maxrat)*mult.sign

cov.element.00 <- cov.element/(maxrat^2)

cov.element.11 <- cov.element*(maxrat^2)

}

# GENERATE REs TO REFLECT BASELINE HETEROGENEITY IN INTERCEPT AT T0

raw.U0 <- rnorm(sampsize.espresso,beta0,sqrt(s2U0-cov.element.00))

# GENERATE REs TO REFLECT BASELINE HETEROGENEITY IN SLOPE AT T1

raw.U1 <- rnorm(sampsize.espresso,beta1,sqrt(s2U1-cov.element.11))

# GENERATE REs TO REFLECT RANDOM MEASUREMENT ERRORS AT T0 AND T1

# THESE MUST BE SIMULATED INDEPENEDENTLY OR THEY WOULD GENERATE A

# COVARIANCE THEMSELVES BETWEEN T0 AND T1

raw.E0 <- rnorm(sampsize.espresso,0,sqrt(s2E))

raw.E1 <- rnorm(sampsize.espresso,0,sqrt(s2E))

# ADD THE RANDOM EFFECTS TOGETHER TO GENERATE THE KEY ANALYTE LEVELS

# ANALYTE AT T0, ANALYTE LEVEL AT T0 WITH MEASUREMENT ERROR

VAL.T0 <- raw.U0+element.00+raw.E0

# ANALYTE LEVEL AT T0 WITHOUT MEASUREMENT ERROR

VAL.T0.noE <- raw.U0+element.00

ANALYTE.TRUE.T0 <- VAL.T0.noE

# ANALYTE AT T1, ANALYTE LEVEL AT T1 WITH ALL ERRORS

VAL.T1 <- raw.E1+raw.U1+raw.U0+element.00+element.11

ANALYTE.OBS.ALL.ERRORS.T1 <- VAL.T1

# OTHER USEFUL QUANTITIES

VAL.T1.noE <- raw.U1+raw.U0+element.00+element.11

VAL.T1.noE.noU0 <- raw.U1+element.11

ANALYTE.OBS.T0 <- VAL.T0

########################################################

# STEP 3 #

########################################################

# CALCULATE KEY VARIANCE COVARIANCE MATRICES. THAT IS

# DERIVE OBSERVED VARIANCE COVARIANCE MATRICES

# WHICH CAN THEN BE COMPARED WITH THEIR THEORETICAL

# EQUIVALENTS TO CONFIRM THAT THIS R CODE DOES GENERATE

# DATA WITH THE CORRECT CHARACTERISTICS

# THE INTERCEPT SLOPE COVARIANCE MATRIX AT THE 2ND LEVEL OF THE

# HIERARCHICAL MODEL

cov.mat.empirical.noE.noU0 <- cov(cbind(VAL.T0.noE,VAL.T1.noE.noU0))

# THE COVARIANCE MATRIX BETWEEN ANALYTE LEVELS AT T0 (INCLUDING ALL ERRORS)

# AND ANALYTE

cov.mat.empirical.full <- cov(cbind(VAL.T0,VAL.T1))

# COVARIANCE MATRIX BETWEEN ANALYTE LEVELS AT T0 (WITH NO MEASUREMENT ERROR)

# AND ANALYTE LEVELS AT T1 (WITH ERRORS ARISING FROM SLOPE HETEROGENEITY)

cov.mat.empirical.noE <- cov(cbind(VAL.T0.noE,VAL.T1.noE))

###########################################################

# COMPARE THEORETICAL AND OBSERVED (WITH ERROR) RESULTS #

###########################################################

# UNIVARIATE DISTRIBUTION OF TRUE VALUES AT T0 WITH NO MEASUREMENT ERRORS

simulated.true <- ANALYTE.TRUE.T0

mean.theory <- beta0

var.theory <- s2U0

# UNIVARIATE DISTRIBUTION OF VALUES AT T1 WITH ALL ERRORS

simulated.with.error <- ANALYTE.OBS.ALL.ERRORS.T1

mean.with.error <- beta0+beta1

var.with.error <- s2U0+s2U1+s2E+2*covU01

# FIXED COEFICIENT MODEL

fixed.coefs.theoretical <- c(beta0,beta1)

ANALYTE.vect <- c(ANALYTE.OBS.T0,ANALYTE.OBS.ALL.ERRORS.T1)

TIME.vect <- c(rep(0,length(ANALYTE.OBS.T0)),rep(1,length(ANALYTE.OBS.ALL.ERRORS.T1)))

glm.mod <- glm(ANALYTE.vect~TIME.vect)

fixed.coefs.as.simulated <- summary(glm.mod)$coefficients[,1:2]

# COVARIANCE U0:U1 NO s2E ERROR

cov.mat.U0U1.theoretical <- cbind(c(s2U0,covU01),c(covU01,s2U1))

dimnames(cov.mat.U0U1.theoretical) <- list(c("VAL.T0.noE","VAL.T1.noE.noU0"),

c("VAL.T0.noE","VAL.T1.noE.noU0"))

# COVARIANCE T0:T1 WITH ALL ERRORS

theoretical.var00 <- s2U0+s2E

theoretical.var11 <- s2U0+s2U1+s2E+2*covU01

theoretical.cov01 <- covU01+s2U0

cov.mat.T0T1.theoretical <- cbind(c(theoretical.var00,theoretical.cov01),

c(theoretical.cov01,theoretical.var11))

dimnames(cov.mat.T0T1.theoretical) <- list(c("VAL.T0","VAL.T1"),c("VAL.T0","VAL.T1"))

#####################

# SUMMARIZE RESULTS #

#####################

cat("\n\n\nUNIVARIATE DISTRIBUTION OF ANALYTE VALUES AT T0 WITH NO MEASUREMENT ERRORS\n--\n\nTHEORETICAL\n mean variance\n",

" ",mean.theory," ",var.theory,"\n\nAS SIMULATED\n mean variance\n",

" ",mean(ANALYTE.TRUE.T0)," ",var(ANALYTE.TRUE.T0),"\n\n")

cat("\n\n\nUNIVARIATE DISTRIBUTION OF ANALYTE VALUES AT T1 WITH ALL ERRORS\n-------------------\n\nTHEORETICAL\n mean variance\n",

" ",mean.with.error," ",var.with.error,"\n\nAS SIMULATED\n mean variance\n",

" ",mean(ANALYTE.OBS.ALL.ERRORS.T1)," ",var(ANALYTE.OBS.ALL.ERRORS.T1),"\n\n")

cat("\n\n\nFIXED COEFFICIENT ESTIMATES\n---------------------------\n\nTHEORETICAL\nintercept slope\n",

fixed.coefs.theoretical,"\n\nAS SIMULATED\nintercept slope (with SEs)\n",

fixed.coefs.as.simulated[1,1]," ",fixed.coefs.as.simulated[2,1],"\n",

fixed.coefs.as.simulated[1,2]," ",fixed.coefs.as.simulated[2,2],"\n")

cat("\n\n\n\nCOVARIANCE U0:U1 (Covariance Structure at Level 2 [Date of Birth] in MLwiN)

----------------------------------------------------------------------------\n\nTHEORETICAL\n")

print(cov.mat.U0U1.theoretical)

cat("\n\nAS SIMULATED\n")

print(cov.mat.empirical.noE.noU0)

cat("\n\n\n\nCOVARIANCE T0:T1 (BETWEEN TIME 0 AND TIME 1 VALUES - WITH ERRORS

----------------------------------------------------------------\n\nTHEORETICAL\n")

print(cov.mat.T0T1.theoretical)

cat("\n\nAS SIMULATED\n")

print(cov.mat.empirical.full)
